# Being in a safe and thus secure place, the core of early labour: A secondary analysis in a Swedish context

**DOI:** 10.3402/qhw.v11.30230

**Published:** 2016-05-10

**Authors:** Ing-Marie Carlsson

**Affiliations:** School of Health and Welfare, department of health and nursing, Halmstad University, Halmstad, Sweden

**Keywords:** Childbirth, early labour, grounded theory, health geography, safe place, secondary-analysis

## Abstract

**Background:**

Early labour is the very first phase of the labour process and is considered to be a period of time when no professional attendance is needed. However there is a high frequency of women who seek care at the delivery wards during this phase. When a woman is admitted to the delivery ward, one role for midwives is to determine whether the woman is in established labour or not. If the woman is assessed as being in early labour she will probably then be advised to return home. This recommendation is made due to past research that found that the longer a woman is in hospital the higher the risk for complications for her and her child. Women have described how this situation leaves them in a vulnerable situation where their preferences are not always met and where they are not always included in the decision-making process.

**Aim:**

The aim of this study was to generate a theory based on where a woman chooses to be during the early labour process and to increase our understanding about how experiences can differ from place to place.

**Methods:**

The method was a secondary analysis with grounded theory. The data used in the analysis was from two qualitative interview studies and 37 transcripts.

**Conclusion:**

The findings revealed a substantive theory that women needed *to be in a safe and thus secure place* during early labour. This theory also describes the interplay between how women ascribed their meaning of childbirth as either a natural live event or a medical one, how this influenced where they wanted to be during early labour, and how that chosen place influenced their experiences of labour and birth.

The early labour process, meaning the very early phase of the first stage of labour, is a period of time in the birth process when no professional attendance is thought to be needed (McIntosh, 2013). Therefore, in Western countries women are advised by midwives in antenatal care to remain in their homes during this phase until labour is established (Green, Spiby, Hucknall, & Richardson Foster, 2012). This means that most women and their partners are left to themselves during early labour. However, women may feel that it is difficult to manage this phase alone, and they have described the early labour phase as a situation that is complex and unknown (Eri, Bondas, Gross, Janssen, & Green, 2014). Facing an unknown or little-known situation may cause stress to the pregnant woman and her partner. Research findings about the early labour phase describe how women often feel uncertain (Eri, Blystad, Gjengedal, & Blaaka, 2010) and lack confidence in how to handle the situation (Cheyne et al., 2007). Despite advice to remain at home there is a high frequency of women who seek out care at the delivery wards during this phase. In a retrospective study, Lundgren, Andren, Nissen, and Berg (2013) reported that as many as 17.6% pregnant Swedish women sought care during early labour due to contractions. The frequency has been reported even higher in other countries (Bohra et al., 2003; Jansen, 2006).

## Background

The concept of an “early” or “latent” phase of labour is a construction made by professionals, for professionals. This construction breaks down the birth process into stages and phases; the construction underpins diagnosis and thereby informs decisions made in obstetric care (Friedman, 1962; Friedman & Sachtleben, 1961; NICE, 2007). The diagnosis and distinction between early or established labour relies on women's contractions but is primarily judged on the clinical assessment of cervical dilatation made by the midwife or obstetrician. A woman who spends her time at home during early labour must rely on her own experience of labour contractions without any examinations made. Therefore, one reason why women seek care at hospitals during the early labour process is because they are uncertain about how advanced their labour is and whether it is established (Beebe & Humphreys, 2006; Carlsson, Hallberg, & Odberg Pettersson, 2009; Cheyne et al., 2007; Dixon, Skinner, & Foureur, 2013). Another reason for a woman to arrive at hospital during early labour is that her partner may prefer that the woman be admitted to the hospital (Cheyne et al., 2007).

In the Western world there is a common policy that if a woman seeks care at the delivery ward and is diagnosed as being in early labour she will be advised to return home and wait until she is further on in the labour process. This recommendation is due to research findings that women admitted to hospital in early labour are more likely to end up experiencing interventions and complications during labour (Bailit, Dierker, Blanchard, & Mercer, 2005; Lundgren et al., 2013; Neal et al., 2014). Admission to hospital at an early stage of labour has also been shown to put women at risk for a higher frequency of caesarean sections (Bailit et al., 2005; Davey, McLachlan, Forster, & Flood, 2013; Rahnama, Ziaei, & Faghihzadeh, 2006; Yang, Hur, & Kim, 2013). Furthermore, admission to hospital also requires more resources, and a recent study concluded that excessive costs could be reduced by delaying admission to hospital until established labour has begun (Tilden, Lee, Allen, Griffin, & Caughey, 2015). Earlier studies also found that women who sought contact with the delivery wards during the early labour process were not always adequately helped by professionals (Eri et al., 2014). For example, Eri, Blystad, Gjengedal, and Blaaka (2011) found that women who phoned the delivery ward were given general advice rather than individualised advice. Likewise, if women choose to seek admission before established labour, then meetings with professionals could be problematic. Research findings indicate that midwives position themselves as a kind of gatekeeper, and that women who seek care feel that they have to negotiate their credibility before they can gain access to the delivery ward (Eri et al., 2010). In addition, women are judged negatively by the midwives if they “come in too early,” and this judgement causes them to feel embarrassed and vulnerable (Eri et al., 2010). Furthermore, when the women's experiences of their contractions are not consistent with the diagnoses from the clinical assessments of cervix dilatation made by the midwives, it could lead them to feeling abnormal (Carlsson et al., 2009; Eri et al., 2010). The fact that midwives use assessment of cervix dilatation as admission criteria leaves women without influence in the process of deciding whether they can remain at the delivery ward (Eri et al., 2010). These issues aside, Lundgren et al. (2013) found that most women who sought care (57.8%) during early labour also remained at the hospital until they had given birth. Reasons for this are unclear, but research has found that women who are sent home during this phase may feel unsupported and dissatisfied and experience increased anxiety (Barnett, Hundley, Cheyne, & Kane, 2008; Green et al., 2012; Scotland, McNamee, Cheyne, Hundley, & Barnett, 2011).

Two earlier qualitative studies have explored women's experiences during the early labour process (Carlsson et al., 2009; Carlsson, Ziegert, Sahlberg-Blom, & Nissen, 2012). The first of these studies explored women who were admitted and remained in the delivery ward during the early labour phase (Carlsson et al., 2009). The findings from this interview study suggested that these women expressed a need to hand over the responsibility for the labour process, to ensure that it was continuing normally, for themselves and for their unborn babies’ safety. The second study (Carlsson et al., 2012) interviewed another group of women—those who chose to remain in their homes until established labour. The results from this study were very different. These women expressed having a sense of power and they were focused on maintaining this power during early labour. The present study arose from these contrasting results and led us to reflect on places and spatial environments rather than considering how to prevent women in early labour from being admitted to hospital. It might be helpful to first explore why women choose places to spend early labour in and how this place affects their labour experience. From a woman's point of view this knowledge could lend a deeper understanding about how the needs of women in early labour can be accommodated by the maternity system.

## Aim

The aim of this study was to generate a theory based on where a woman chooses to be during the early labour process and to increase our understanding about how experiences can differ from place to place.

## Methods

This study is a theoretical study based on a secondary analysis of previously collected data and research findings from two qualitative studies (Carlsson et al., 2009, 2012). According to Glaser (2013) this method of using secondary analysis can be useful since it enables the researcher to construct new findings and clarify past research findings (Glaser, 2013). This study elaborated on two grounded theory studies (Carlsson et al., 2009, 2012). Therefore, the same method was also used as an approach in this study. Another reason for using grounded theory was that it is a suitable method when the purpose is theory development (Glaser & Strauss, 1967).

### Data collection

This secondary analysis involved existing original data from two in-depth interview studies, and the sample consisted of 37 transcripts. Of these transcripts, 18 were sourced from the first study (Carlsson et al., 2009) and 19 from the second study (Carlsson et al., 2012). The primary interviews were made with women who had given birth in Sweden and the interview questions focused on women's experiences and coping strategies during the early labour phase before active labour was established. The data from the two studies was considered comparable with respect to situation, population, and setting. For both of these studies the interviews were performed postnatal within 2 to 6 weeks after delivery in the western part of Sweden, and women with pregnancy complications were excluded from both studies. Both studies included in this secondary analysis have ethical approval (registration number: 90/2005/511 & 2009/29).

### Data analysis

Data analysis was done by using the previously collected data for further analysis. The data analysis followed the steps used in grounded theory with initial, focused, and theoretical coding and memo writing (Charmaz, 2014). All transcribed data from a total of 37 interviews were read through several times to gain an overall picture of the main concerns of the women in the studies. The first step in the analysis was initial coding. Events or actions that responded to this study's purpose were noted in the margin when found. The meaning of the sentence was captured, labelled, and then written in the margin as codes. These codes were kept close to the data and were often written in an active form to capture the actions. Examples of codes were: *seeking safety*, *having bodily trust*, and *surrounding themselves with the familiar*. Questions were asked during the analysis such as, *Why did these women choose to stay either in their home or to go into hospital during the early labour phase?* and *How does the chosen place affect them?*

The second step in the analysis was the focused coding, where codes with similar meanings were clustered with each other to create concepts. Thereafter, similar concepts were grouped together and transformed to a higher level of abstraction. These clustered concepts formed two categories that explained the women's view of childbirth. These two categories were named (1) *labour and birth as a medical event* and (2) *labour and birth as a natural life event*. A core category, *being in a safe and thus secure place*, emerged that highlighted the women's main concerns during the early labour process before labour was established. The third analysis step, the theoretical coding, involved a phase where questions were asked about the material, and I tried to visualize the relationships between the categories and make connections to move the analysis to a more theoretical level (Charmaz, 2012). During this phase, the primary findings from the two studies were included in order to conduct theoretical sampling. Furthermore the theoretical notes that were written throughout the primary studies and throughout this secondary analysis process were included in the analysis phase. These memos captured the thinking process during the analysis and contained ideas about relationships and patterns in the data and the emerging theory.

## Findings

**Figure 1:**
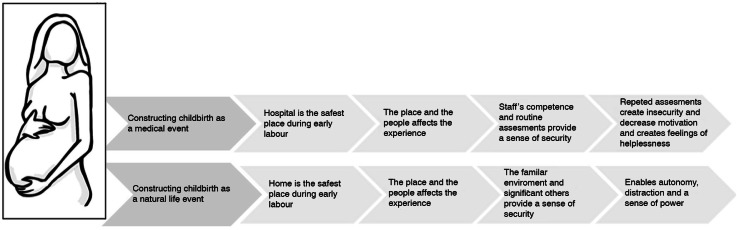
The substantive theory: Being in a safe and thus secure place.

The substantive theory that emerged from the analysis was that the women had a need *to be in a safe and thus secure place* during early labour. The theory also describes an interconnection between how women construct childbirth as either a medical or natural life event and what different geographical space they believe is the safest place to be in during early labour. These beliefs influenced where they wanted to be during early labour and at what point during the labour process they chose to arrive at hospital. Furthermore, the chosen location impacted on their experience of childbirth (Fig. 1).

### The core theory of being in a safe and thus secure place

This core theory describes the overall feeling for the women during the early labour. They solved their main concern, to be safe, by positioning themselves where they believed was a safe and thus secure place for them in this phase of labour. It was important for them to settle down and make themselves a kind of “human nest.” All women in the included studies served as active agents, making thoughtful choices about what they thought was a safe place during that time period. However, the women resolved their main concern differently depending on how they ascribed the childbirth, either as a medical event or a natural life event. Depending on these two different approaches the women were proactive and sought the place they believed was the safest for them to be during early labour. If the women believed that childbirth was a medical event that ought to be controlled and handed over to professionals, then they chose to arrive at hospital during early labour. In contrast, if the women ascribed childbirth as a natural life event, meaning an inherent ability for all women since the beginning of humanity, they remained in their homes. For all women the secure place was intimately associated with the people at that place.

### The hospital as a safe and thus secure place

For those women who viewed labour and birth as a medical event with risks, the hospital was considered the safest place to be during early labour. These women positioned themselves inside the hospital and expressed that the hospital was a safe place due to the staff's competence and that their knowledge provided them with a sense of security. The presence of a caregiver nearby, just outside the door, was an additional factor that contributed to the women's sense of safety. The women used positive words like *to get help if you need it*; *having someone nearby*; *having somebody that can check in on you*; and *being comforted by others*.

For those women who regarded the hospital as a safe place it was important to arrive on time.

It was more like … that it felt safe to be there, at the hospital, to be there on time and just wait (Study I, Participant 11)

Sometimes it was as if the women arrived early at the labour ward just to claim a room, which they could then leave if they wanted. One woman proposed that it would have been optimal if every woman had an assigned room at the maternity ward that they could leave and come back to whenever they pleased.

It was enough to just be present, just being there. There was no specific medical need at that point … although that became important later, but at that time it was just enough being there. (Study I, Participant 18)

Moreover, the birth environment included routines and repeated assessments conducted by midwives, which decreased the women's feelings of insecurity. However as time passed and the assessments with vaginal examinations were repeated, without showing any labour progress, the routines and knowledge of poor labour progress resulted in the opposite effect, increased insecurity. The point at which labour was diagnosed as having no or very little progress was when some women raised concerns about why the process was taking so long and whether their labour really was considered normal. Being aware that “nothing or very little was happening” with the labour progress largely impacted on the women and reduced their motivation to continue with the labour process and to endure the pain. The result was that the labour pain was regarded as unnecessary—why should they have pain without progress? Since these women were in hospital, they expected to be cared for and that something should be done to end the process. When nobody acted, this ended with a feeling of helplessness and they felt they were being inadequately cared for.

The worst part of this situation was that nothing happened. I felt so helpless, please help me … I am in a hospital, do something! (Study I, Participant 3)

In this situation some women described themselves as victims and they felt disempowered and with feelings of hopelessness. What is notable is that the midwives considered the process to be normal since the women were in the latent phase, and later not one of these cases was diagnosed as a prolonged latent phase. In summary, the hospital setting with all the procedures, monitoring, and assessments originally felt secure for the women but they subsequently felt the opposite. The hospital instead became an insecure place and they had a negative experience.

### The home as a safe and secure place

In contrast, the women in the second study who preferred to remain in their homes during early labour considered birth to be a natural life event that woman throughout history have experienced. For them, the home was the natural place to be in and they remained in their homes until established labour. These women had heard positive stories; they often referred to their mothers and sisters who had an easy time giving birth and mentioned that this was probably inherited.

I can do this, my body is made for this and women have done this throughout history. I will survive and I can do this, I never had any doubt. (Study II, Participant 11)

These women used their homes as a kind of base camp, a safe place that they could leave and return to whenever they pleased. This private sphere was a sheltered place where they could regain strength and get some rest. They seemed to surround themselves with the environment by choosing a place in their home where they were comfortable, for example cuddling on the sofa and with people who were significant to them.

Yes home is home, you know what you have … my own bed and sofa, and food and all that. I feel safe here; I am surrounded by my own things. You have more freedom, like if you want to go out for a bit and things like that. (Study II, Participant 8)

The home as a safe place also gave them the freedom to choose significant others with whom to surround themselves and share the experience. Friends and family were seen positively and they used them to distract themselves, especially before they were in too much pain. Later on, the women described how it was critical that their partner be near them, not necessarily in the same room but nearby so that they could call them if they needed them. Their position in the home enabled autonomy and mobility and hence distraction, and this contributed to their ability to be in the present.

These two groups of women had quite polarized experiences of their early labour. Compared with those who spent their early labour at the delivery ward, these women never doubted their own bodies or that labour was normal. It is of note that they did not have any examinations of their cervix dilatation and therefore did not know if labour was progressing or not. They never mentioned feeling disempowered or hopeless; instead they described themselves as powerful. Furthermore, they never doubted their own ability to manage the labour and they felt that they had a power within that helped them to cope during this stage. Maintaining this power was the central focus for these women and this involved a sense of authority over their own bodies.

## Discussion

The results of this study lead us to reflect on the significance of the spatial environment where early labour takes place. The human being and her environment is one of the core concepts in nursing science (Fawcett & Alligood, 2005). In this article the environmental concept is widened and includes the concept of place, influenced by the discipline of health geography (Andrews, 2002). Research within the geography of health suggests that a place is a location that is constructed through the interactions between people and the physical environment (Andrews, 2002). Furthermore, the place also constitutes what comes about (Kearns & Joseph, 1993). Findings from this study revealed that there was an interconnection between how women constructed childbirth, as either a medical or natural life event, and what different geographical space they believed was the safest place to be in during early labour. In turn, these beliefs influenced at what point during the labour process they chose to arrive at hospital.

These findings are in accordance with a previous study by Rämgård (2006), who found that when a woman is facing momentous changes like pregnancy and childbirth she seeks places that represent security. In this study one significant place that was appraised as safe was the delivery ward at the hospital. This is similar to a study by Green et al. (2012), which found that the hospital gave “the women a sense of safety.” In their study a sense of safety was channelled through a phone call with a midwife, which made the women comfortable with remaining at home longer, provided they were reassured that they were welcome and allowed to come in. When the place is an institution, in this case a hospital, it is intimately connected with the care that takes place within (Teather, 1999). From a spatial perspective, places are not just physical but are also given meaning to by people, and people also have feelings attached to places (Crang, 2008).

Dixon, Skinner, and Foureur (2014) concluded that it seems as if women have an inherent need to move to a safe place during the labour process. In the first study included here the women chose to move to the delivery ward during early labour. During the 1900s, the development of social institutions increased and have since become more and more associated with trust and safety (Giddens, 1990). This trust is reflected and embedded in the social space that is constructed by the perceptions, opinions, beliefs, and norms of society (Davis-Floyd, 2003; Giddens, 1990; Gren, 2003). In addition to the increased trust in social institutions, late modernity has increasingly come to adopt risk thinking. This way of thinking, that every risk must be eliminated, could influence and impact on how women construct childbirth. It could also influence how women and their partners seek care during the labour process and how decisions are made by professionals about their care (Giddens, 1990; Regan & McElroy, 2013). Risk is related to security and safety and also to responsibility (Giddens, 1999). The first of the two studies included in this secondary analysis (Carlsson et al., 2009) had a core category labelled *handing over responsibility to the professionals*. Paradoxically, handing responsibility over to professionals might be more risky than safe. Today delivery wards are often places where medical care is dominant. They are characterized by extensive monitoring, assessments, and interventions used to eliminate risks, even to those women who are staying there during early labour (Bailit et al., 2005; Neal et al., 2014). Scamell (2011) found that midwives tell expectant parents that childbirth is a normal event, while at the same time they act as if childbirth is a risk. I argue that, in this way, having several vaginal examinations during early labour was a factor in the women's decreased motivation. These examinations made the women aware of their slow progress; discussions about time frequently reoccurred in the interviews. As time passed and no progress occurred they began to lose hope and began to doubt that their bodies would be able to handle giving birth. This secondary analysis highlights that place constitutes the situation and that caregivers and their actions are entwined with place. It was obvious that the disempowerment the women felt from the vaginal examinations, which made them lose trust in their bodies, also decreased their motivation and made them feel like victims. This type of negative experience can decrease women's childbirth self-efficacy and have a negative effect on women's mental health and well-being (Fenwick, Gamble, & Mawson, 2003). Feelings of being a victim arise when you cannot influence your situation; Fahy and Parrat (2006) claimed that women need a birth territory that gives them power to do what they want during the labour process. This is in accordance with the narratives from the second study (Carlsson et al., 2009). These women felt that their home was a familiar environment that gave them the opportunity to be mobile and autonomous, and this made it easier for them to maintain their power, which helped them cope during this stage of labour. Their experiences were intrinsically linked with the women's bodily perceptions and their childbirth self-efficacy. They never doubted that they had the capability or that they were able to go through the birth process. Furthermore, these women were not victims; instead they were empowered throughout this process.

In the debate about managing childbirth, we must not forget to include early labour. This period is a sensitive phase that might affect the birth as a whole (Wuitchik, Bakal, & Lipshitz, 1989). The findings from this study have increased our knowledge about childbirth and how women's views on childbirth influence their decision on where to be during early labour. This view is probably grounded in their society and history and we may assume that these decisions are made unconsciously. To turn this decision around is difficult and a huge undertaking; therefore it is preferable to meet the woman's preferences to be in a safe place. If this means that she will spend early labour at the delivery ward then the midwife should make sure that she is met in a welcoming manner and ensured individual care (Ängeby, Wilde-Larsson, & Sandin-Bojö, 2015). Likewise, this care should be good care where professionals mitigate interventions and promote women's agency and well-being. However, it is difficult for midwives to respond to each woman's individualised request if the delivery setting suffers from staffing shortages and a lack of beds. Thus this is also an issue at the organization level. These findings highlight that midwives and obstetricians need to understand that place of care matters to their patients. Perhaps it is time to reintroduce the so-called rest room located outside the delivery ward, which was used for therapeutic rest. Placing the woman outside but nearby the delivery ward informs the woman, her partner, and the staff that established labour has not yet begun and the situation should be treated as if the woman were still at home, but with the possibility of support from a midwife. There is an ongoing debate about place of birth, although little attention has been paid to the place of early labour. Looking at how places influence women is one way of understanding how to create environments that promote health and well-being (Atkinson, Fuller, & Painter, 2012).

There are several limitations to this study that should be taken into consideration. First, the included studies in the secondary analysis are both from the same western part of Sweden with a quite homogenous sample. However, this is also a strength of the method, since it is preferable to use material that is comparable when conducting a secondary analysis (Glaser, 2013). This study emphasises that people construct their reality and that this behaviour is embedded in our society and culture. The findings presented in this article reflect the culture in Sweden, but I argue that the findings are transferrable to other, similar contexts and groups of women (Hallberg, 2013). Other researchers have found similar results in material collected in Norway (Rämgård, 2006) and the United Kingdom (Green et al., 2012). It could be argued that the findings could have been influenced by several factors other than place, such as psychological factors, personality, or the women's physical attributes. However, it should be kept in mind that grounded theory as a method creates theories about how people interpret reality rather than statements about reality itself (Glaser & Strauss, 1967).

## Conclusion and implications

This study has provided insight into early labour and the women's main concern during this period of time in the labour process. The secondary analysis found that the core need for women during early labour is to be in a safe and thus secure place and that places influence women's experiences. In summary, the findings from the analysis of the included papers separated the women's experiences depending on where the women spent their early labour. The findings highlight the issue of how to manage those women who arrive at hospital during early labour. It's important that such women are helped and given an opportunity to feel safe and secure, rather than being managed as if they were in established labour. Repeated measurements and vaginal examinations are devastating for a woman's motivation and her belief in the ability of her own body to give birth. The challenge for clinical settings is to recognize a woman's preferences, take them into account, and enable her to participate in decision making about her own care. Therefore, it would help to separate the physical space at the maternity ward where women in early labour are waiting. This action could communicate to both women and professionals that early labour should be allowed to proceed with minimal interference. Last, further research is needed into how women's agency and autonomy can be maintained while they are in hospital.
